# Fluorination of antimonene hexagons†

**DOI:** 10.1039/d4cc03423f

**Published:** 2024-09-16

**Authors:** Michael Fickert, Rebeca Martinez-Haya, Diego López-Alcalá, Frank Hauke, José J. Baldoví, Andreas Hirsch, Gonzalo Abellán

**Affiliations:** a Department of Chemistry and Pharmacy and Joint Institute of Advanced Materials and Processes (ZMP) Friedrich-Alexander-Universität Erlangen-Nürnberg (FAU) Fürth 90762 Germany; b Instituto de Ciencia Molecular (ICMol) Universidad de Valencia Valencia 46980 Spain gonzalo.abellan.uv.es

## Abstract

Fluorination of two-dimensional (2D) antimonene hexagons synthesized through a colloidal bottom-up approach has been explored using microwave-induced plasma and reactive ion etching fluorination strategies through the generation of CF_4_. The stability of the fluorine bond has been corroborated through DFT calculations. This work paves the way for further halogen-derivative modifications of heavy 2D pnictogens.

## Abstract 2

Fluorination of two-dimensional antimonene hexagons synthesized through colloidal synthesis has been reached using CF_4_ plasma through a reactive ion etching process.

## Graphical abstract

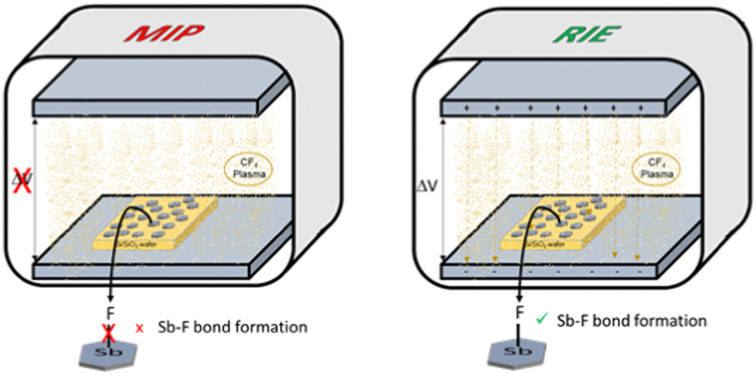

Since its prediction in 2015^1^ and further isolation in 2016,^2^ interest in antimonene has increased due to its unique atomic structure (as a quasi-van der Waals material)^3–5^ and its chemical flexibility, which enables exotic (inter-allotropic) phase engineering.^6,7^ This is of great significance in applications beyond electronics, encompassing fields like biomedicine, sensing, energy, and catalysis.^6^ In this sense, one of the most exploited strategies to modulate and boost the properties of 2D materials is interface functionalization, mainly studied in graphene or even other pnictogens such as phosphorene.^8–10^ However, the functionalization of antimonene has been barely explored since its use is still in its infancy.

More specifically, the first experimental study was reported in 2017 by Abellán *et al.* using perylene diimide and resulting in a charge-transfer functionalization.^11^ Lately, to the best of our knowledge, few other noncovalent^12,13^ and covalent^14–17^ functionalization works have been reported. Among all selected reagents used for covalent functionalization, halogens are particularly interesting for tuning the band gap or increasing the stability of 2D materials against degradation.^18,19^ Indeed, their notable electronegativity allows them to form bonds with a wide range of metallic and nonmetallic atoms. Gases like F_2_ or XeF_2_ or liquid HF_aq_ have been widely used since their high reactivity promotes fluorination on almost any substance or material. Unfortunately, their intrinsic toxicity along with the requirement for special handling techniques and equipment, encourages the study of safer methodologies.^20^ This scenario drives the opening of the chemical toolbox to find adequate reaction conditions to fluorinate antimonene. In this regard, the use of plasma can reach high radical density and selectivity at low temperatures and mild voltages, preventing unwanted diffusion and degradation of the functionalizing material. Indeed, plasma treatments typically have a minimal effect on materials, which is crucial for applications in the microelectronics industry.^21,22^ Regarding antimonene, it should be highlighted that synthetic procedures lacking morphological control may generate an amorphized material susceptible to oxidation and subsequently poorly halogenated.^17^ Herein, taking advantage of newly developed protocols to produce ultrathin hexagonal samples of antimonene with a high-quality structural control,^23,24^ we explored the fluorination of antimonene hexagons using two reactive systems based on CF_4_ plasma (Scheme 1) with optimum results.

**Scheme 1:**
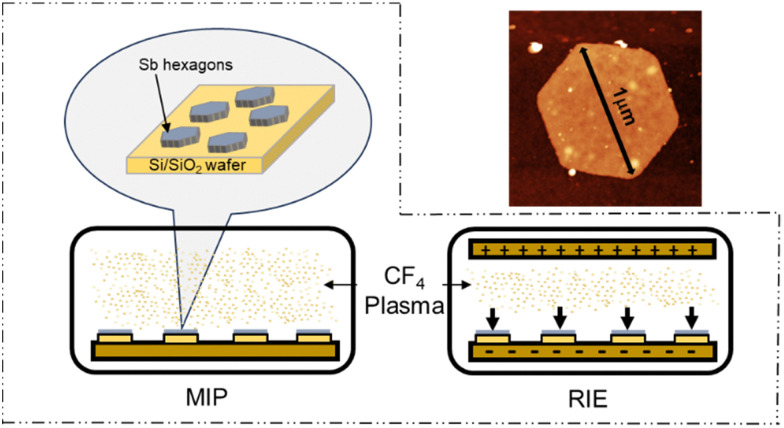
(a) Illustration of the CF_4_ plasma treatment using microwave-induced plasma treatment (MIP) and reactive ion etching (RIE) over Sb hexagons deposited on a Si/SiO_2_ wafer. (b) AFM image of an Sb hexagon flake of *ca.* 1 μm in lateral dimensions.

The methodology of this study paves the way for the halogenation of antimonene with a controlled morphology, expanding the scope of covalent functionalization routes and opening the door to other 2D pnictogen functionalization. Prior to carrying out the experimental procedures, we first gained insights into the viability of our approach regarding F–Sb bond formation in the ultrathin hexagonal antimonene flakes. Thus, DFT calculations were performed to evaluate the bond stability, its nature, and the importance of the Sb/F ratio. To carry out this task, first, the band structures with atomic and orbital contributions of different antimonene systems were determined.

Our calculations indicate that pristine Sb monolayer has a semiconductor ground state with a direct band gap of ∼1 eV (Fig. S1a, ESI†). Then, we constructed a 3 × 3 × 1 supercell of Sb monolayer and placed F atoms on one surface of the system. To consider different fluorination ratios we constructed three different systems, namely Sb_18_F_3_, Sb_18_F_6_ and Sb_18_F_9_ with 0.16, 0.33 and 0.5 F/Sb ratio, respectively. Their electronic band structures with atomic and orbital contributions are shown in Fig. 1b and Fig. S1b–d (ESI†). Moreover, we observe that the fluorination of the Sb monolayer causes the energy levels involved in the Sb–F chemical bonds to be placed now in the Fermi level. These bands have a hybridized character between the p_*z*_ orbitals of F and the Sb atoms, and their intensity increases at higher F/Sb values, supporting the idea of a robust chemical Sb–F bond. Moreover, we observe that fluorination of Sb monolayers causes the energy levels of Sb atoms to be shifted towards higher energies as the F/Sb ratio increases. This is due to the acceptor nature of F atoms, whereas the intrinsic band gap of ∼1 eV in Sb bands remains almost intact. In other words, the F/Sb ratio increases the intensity of the hybridized p_*z*_ levels around the Fermi level due to a larger presence of Sb–F interactions, but there are no further changes in the electronic structure of the functionalized system when the Sb/F ratio increases. Therefore, we can conclude that the F/Sb ratio does not play an important role in the covalent functionalization of Sb monolayers. To further confirm the presence of Sb–F chemical bonds, we computed the charge distribution of the Sb_18_F_2_ monolayer (Fig. 1a and Fig. S1, ESI†) where an out of plane accumulation can be observed between the Sb and F adsorbed atoms in the region of the chemical bond. Voronoi charge analysis revealed that electronic density flows towards the F atoms (+0.2 and −0.27 *e* atomic charges in Sb and F, respectively) supporting the idea of a chemical interaction and the stability of covalent functionalization of Sb *via* fluorination.

**Fig. 1:**
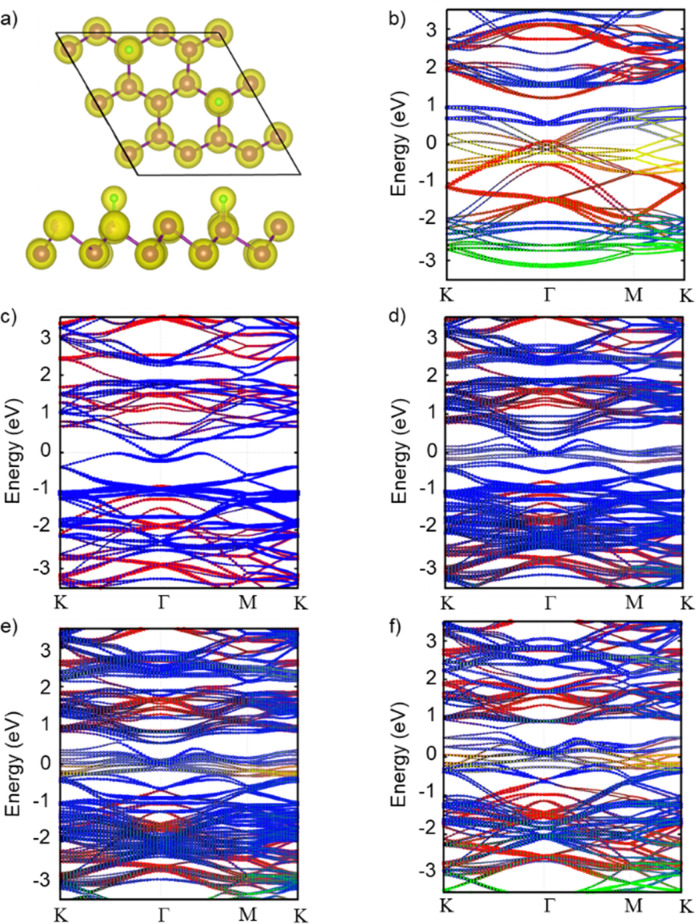
(a) Top and side view of Sb_18_F_2_ monolayer charge density. (Isosurface set to 0.08 *e* Å^−3^. Color code: purple (Sb) and green (F). (b) Band structure with atomic and orbital contributions for Sb_18_F_9_ and for an AB stacked β-Sb bilayer Sb_36_ (c), Sb_36_F_3_ (d), Sb_36_F_6_ (e) and Sb_36_F_9_ (f). Colour code: red (Sb-p_*x,y*_), blue (Sb-p_*z*_), green (F-p_*x,y*_) and yellow (Sb-p_*z*_).

Following the same methodology, we explored a similar scenario in a bilayer β-Sb. First, we investigated the different stacking possibilities in the bilayer system. Fig. S2 (ESI†) shows the calculated electronic band structure in the eclipsed (AA) and slipped (AB) stacking of β-Sb, respectively. AA stacking shows a small indirect electronic band gap whereas in the AB stacking a hole pocket is crossing the Fermi level and a metallic ground state is found. The AB stacking has been shown to be the most stable configuration with an energy of 0.038 eV per atom lower than the AA configuration. This vanishing of the electronic band gap from the monolayer to the bilayer is compatible with previous theoretical and experimental studies.^25,26^ Furthermore, we explored the effect of monotopic fluorination on the antimonene bilayer, as it can be a powerful method to tune the band gap of these materials. Fig. 1c–f shows the evolution of the electronic band structure with orbital-resolved atomic contributions upon fluorination in the same F/Sb ratios as in the monolayer case. The addition of F atoms to one side of the bilayer system generates the same effect as in the monolayer, where the hybridized Sb–F p_*z*_ bands are placed at the Fermi level. This also supports the idea of a possible fluorination of β-Sb bilayers as we have demonstrated for the monolayer. Once the stability of the bond over the antimonene hexagons was clearly established, we proceeded with the experimental part using CF_4_ plasma. The use of CF_4_ plasma is a highly efficient method for surface fluorination due to its high reactivity, with the reaction occurring predominantly from one side, or monotopically, allowing better control over the reaction times. Additionally, the reaction is thickness-dependent: the fewer the number of layers, the higher the reactivity.^27^ Therefore, we took advantage of our optimised bottom-up colloidal synthesis^23^ for the production of highly anisotropic ultrathin hexagonal antimonene using chloroform as a solvent through the process to minimise surface oxidation, see Section S1 of the ESI.†.^23^ Those antimonene nanosheets (Sb NS) were characterized by AFM and Raman spectroscopy prior to their fluorination, see Section S4 of the ESI† and Scheme 1b. Briefly, the AFM characterization reveals well-defined hexagons with lateral dimensions in the range of 0.6–1.2 microns and heights in the range of 7–20 nm with a relatively smooth surface. Regarding the Raman spectrum, bulk antimony depicts two main phonon peaks: A_1g_ and E_g_ modes at 149.8 and 110 cm^−1^, which correspond to the in-plane and out-of-plane vibrational modes, respectively. These bands are shifted towards higher wavenumbers as the number of layers decreases.^6^ Fig. S3 (ESI†) shows two well-defined vibrational modes at 125 and 161 cm^−1^ that perfectly match with a few layers of antimonene.^28^ No signals at 190.6 and 255.2 cm^−1^ related to Sb_2_O_3_ can be detected in the spectrum.^29^ Subsequently, two distinct fluorination methods utilizing CF_4_ plasma were used: microwave-induced plasma (MIP)^30^ and reactive ion etching (RIE).^31^ With MIP, a sample is placed in an ultrahigh vacuum chamber, then, CF_4_ gas is introduced into the chamber where microwave energy generates the plasma and ignites directly around the sample (Scheme 1). This allows the isotropical formation of reactive ions in the chamber and around the sample, potentially leading to a reaction with Sb. In turn, RIE employs a similar approach but introduces an electrical field that induces an anisotropic trajectory over the ionized atoms, accelerating them towards the surface of the sample (Scheme 1).^32^ The introduction of this physical component together with the chemical one, significantly enhances reactivity. This typically results in well-defined features with high selectivity, allowing for the etching of specific materials while leaving others intact. Actually, due to the high controllability of the method, it is commonly employed in microelectronics for etching the surfaces of Si wafers.^33^

Inasmuch as functionalization can exclusively take place on the surface of the hexagonal Sb nanosheets, XPS measurements were conducted to demonstrate the successful covalent fluorination. For this, MIP, RIE and pristine hexagonal Sb samples were measured (Fig. 2 and Section S5, ESI†). The F 1s and the C 1s regions were considered to analyse the F–Sb bond formation instead of the Sb region, as the Sb region overlaps with the O one and the signal interpretation may be less clear due to the out of scale Si–O peak inherent to the wafer. Since the Sb nanosheets did not fully cover the whole Si/SiO_2_ wafer surface and the Sb signals are very weak in comparison with the intense O 1s signal, it is difficult to assign Sb peaks, as they overlay with the O ones. Fig. S4 (ESI†) corresponds to pristine Sb NS prior to functionalization, and thus no fluor signal can be observed, whilst the binding energies for sp^3^ hybridized C atoms, a C–O and a C=O can be found at 284.8 eV, 286.6 eV and 287.6 eV, respectively. These carbon signals are attributed to adventitious carbon due to unavoidable contamination of the sample under air exposure.^34^ Next, the XPS measurements of the samples obtained with the MIP procedure were evaluated, see Fig. 2a and b. Thus, in Fig. 2a, a single signal is detected at 688.5 eV corresponding to a C–F bond. The absence of any other F 1s signal reveals the presence of only one fluorine species. Regarding the C 1s region of this sample (Fig. 2b), one can observe the adventitious carbon signals, as in the pristine sample (sp^3^–C at 284.8 eV, C–O at 286.4 eV, C=O at 287.5 eV and O–C=O at 289.4 eV). Besides, four new signals attributed to the binding energies of carbon fluoride bonds arise at 288.5 eV, 291.0 eV, 293.2 eV and 295.1 eV, which correspond to the binding energies of C–F, CF_2_, CF_3_ and CF_4_, respectively. Those binding energies agree well with fluorinated graphene samples treated with CF_4_ plasma.^35,36^ This is in good agreement with the XPS spectrum at the F 1s region confirming that only a C–F bond is present in the sample, and attributed to precipitated CF_4_ reagent and fragments of decomposed CF_4_ due to the plasma treatment on the wafer's surface during the process. This reveals that the MIP procedure does not promote the pursued Sb–F bond formation and it may be due to a low reactivity of the CF_4_ plasma that only achieves a surface passivation of the Sb NSs. Subsequently, the RIE method was tested since this technique reaches higher ion energies due to the applied bias voltage. Thus, highly anisotropic etching profiles are achieved, meaning that etching occurs predominantly in the vertical direction,^37^ see Scheme 1 and Fig. 2c and d. In the C 1s region of Fig. 2d one can observe the adventitious carbon signals of C–C, C–O, C=O and O–C=O bonds at 284.8 eV, 286.4 eV, 287.7 eV and 289.3 eV, respectively. Besides, the peak corresponding to the binding energy of CF_2_ can be deconvoluted at 291.8 eV. To our delight, no signal for the presence of CF_4_ can be found, suggesting that most of the CF_4_ plasma reacted with the sample. The F 1s region shows three distinct signals (Fig. 2c). The first one at 689.1 eV is attributed to the binding energy of C–F, like the MIP sample; however, the signal is significantly lower, representing the smallest peak in the region. Two additional signals at lower binding energies are observed, indicative of a metal-fluorine bond. The most prominent peak, at 686.5 eV, is attributed to the formation of a Si–F bond derived from the uncovered area of the Si/SiO_2_ wafer. Indeed such wafers etched with fluorine exhibit a signal at 687.1 eV.^38^ Furthermore, due to the ultrathin nature of the hexagons and their inhomogeneous disposition in the wafers, the high resolution Sb region (Fig. S5, ESI†) reveals a prominent peak related to SiO_2_, which is overlapped with the Sb signals. In any case, deconvolution yields typical peaks associated to Sb–F bonds around 531.5 eV.^39^ Finally, the F 1s signal at 685.2 eV, attributed to the Sb–F bond, in good agreement with previously published results on fluorine-containing Sb compounds,^40,41^ confirms the successful covalent functionalization of hexagonal Sb nanosheets.

**Fig. 2:**
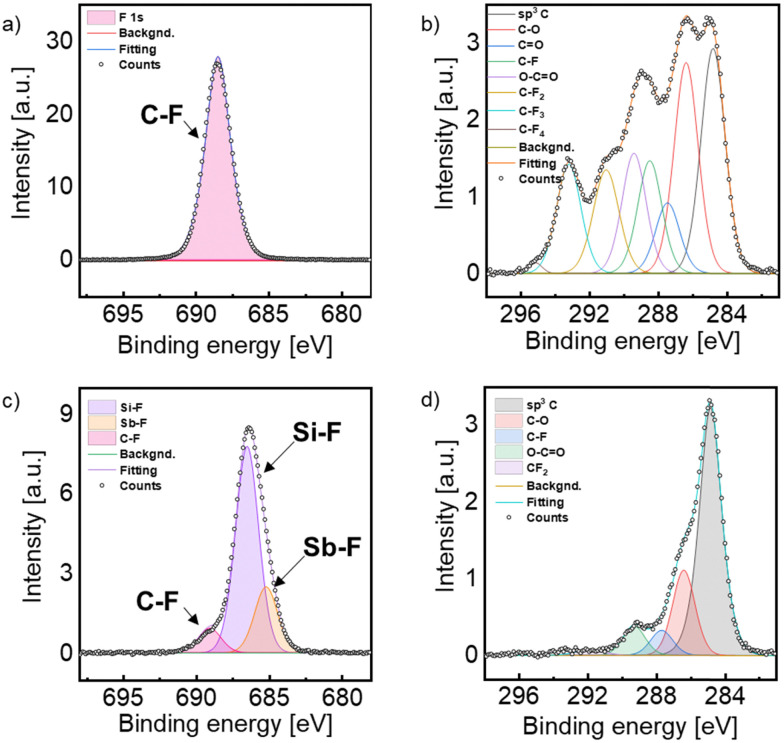
XPS spectra of the Sb hexagons treated with MIP (a) and (b); and RIE (c) and (d) at the F (left column) and the C (right column) binding energy regions.

In conclusion, we have successfully demonstrated a controlled route for the fluorination of high-quality Sb nanosheets using reactive ion etching CF_4_ plasma. This work represents one of the first reports of covalent functionalization of antimonene through a methodology that provides materials with well-defined morphologies compatible with microelectronic devices. This research opens the door for further halogen-derivative functionalization of heavy 2D pnictogens and their heterostructures.

R. M.-H and M. F. contributed equally as first authors. G. A. conceptualized the idea and supervised the project. M. F. performed the experimental part supervised by G. A., R. M.-H., F. H. and A. H. The computational part was performed by D. L. under the supervision of J. J. B. R. M.-H. and M. F. analysed the data. R. M.-H. wrote the manuscript supervised by G. A. All authors discussed the results and contributed to the final manuscript.

This work has been supported by the European Union (ERC-2018-StG-804110-2D-PnictoChem, ERC-Proof of Concept Grant 101101079-2D4H2 & ERC-2021-StG-101042680 2D-SMARTiES), and it is part of the projects PID2022-143297NB-I00, PDC2022-133997-I00 and Excellence Unit María de Maeztu CEX2019-000919-M founded by the Spanish MCIN/AEI/10.13039/501100011033, and the Generalitat Valenciana (CIDEGENT/2018/001 and CIDEXG/2023/1). G. A. is thankful for support by the Deutsche Forschungsgemeinschaft (DFG; FLAG-ERA AB694/2-1) and the FAU (Emerging Talents Initiative grant #WS16-17_Nat_04). R. M.-H. acknowledges the grant FJC2021-047262-I funded by MCIN/AEI/10.13039/501100011033 and by “European Union NextGenerationEU/PRTR”.

## Data availability

Experimental data files supporting this work are openly available at https://doi.org/10.5281/zenodo.13384155 located at the ZENODO repository.

## Conflicts of interest

There are no conflicts to declare.

## Supplementary Material

CC-060-D4CC03423F-s001
