# Supplementary material for: Fluorination of antimonene hexagons
Source: Chem Commun (Camb). 2024 Sep 16;60(91):13336–9. doi: 10.1039/d4cc03423f (PMC11446181; doi:10.1039/d4cc03423f)
Supplement: CC-060-D4CC03423F-s001 [file CC-060-D4CC03423F-s001.pdf]

Supplementary Information (SI) for ChemComm.
This journal is © The Royal Society of Chemistry 2024

Supporting information

Fluorination of antimonene hexagons

Michael Fickert,‡[a] Rebeca Martinez-Haya, ‡[b] Diego López-Alcalá,[b] Frank Hauke,[a] Andreas Hirsch,[a] José J. Baldoví,[b] Gonzalo Abellán,*[b]

a. Department of Chemistry and Pharmacy and Joint Institute of Advanced Materials and Processes (ZMP) Friedrich-Alexander-Universität Erlangen-Nürnberg (FAU) Fürth 90762, Germany.

b. Instituto de Ciencia Molecular (ICMol) Universidad de Valencia, Valencia 46980, Spain.

‡ RM-H and M.F. contribute equally as co-first authors.

1. Materials and general methods…… S1
1.1. Chemicals…… S1
1.2. Methods…… S1
2. Instrumentation…… S2
3. DFT calculations …… S3
4. Sb hexagons characterization…… S4
5. XPS characterization …… S5
6. References…… S7

1. Materials and general methods

1.1. Chemicals

$SbCl_3$,1-octadecene (ODE), 1-dodecanethiol (DDT), oleylamine (OA) and chloroform ($CHCl_3$) were purchased by Sigma Aldrich.

Propan-2-ol (> 98 % purity) and acetone (> 99 % purity) were purchased from VWR.

Si/SiO_2 substrates with an oxide layer thickness of 300 nm was bought and cut into 10 x 10 mm wafer by the "Fraunhofer-Institut für Integrierte Schaltungen IIS" in Erlangen. Additionally, the substrates were bath sonicated in acetone for 5 min and in isopropanol for 5 min. Subsequently, the Si/SiO_2 wafer were dried using an argon flow.

1.2. Methods

1.2.1. Synthesis of Sb hexagonal nanosheets:

The Sb hexagonal nanosheets (Sb NS) were prepared according to a two-step procedure reported in literature [1,2], briefly:

STEP 1: Preparation of the Sb-DDT precursor:

Vacuum was applied to a 50 mL nitrogen round flask with a cock. Afterwards, the flask was heated with a heat gun, after which the flask was filled with Argon. These steps (evacuating/heating/refilling with argon) were repeated three times to remove all residues of water and oxygen in the flask. After cooling down to room temperature $SbCl_3$ (0.91 g, 4 mmol), ODE (6 mL) and DDT (4 mL) were added. The flask was sealed with a septum and heated to 110 °C while stirring continuously. Then vacuum was applied for two hours to degas the chemicals. Afterwards, argon was introduced in the flask and the temperature was increased to 150°C to let all DDT react with $SbCl_3$. At this point, the precursor solution has a yellow color. After cooling down to room temperature, the precursor precipitates out of the solution as a white solid. Therefore, the mixture must be heated to minimum 60 °C until all of the Sb-DDT precursor is dissolved again before it can be used for further reactions.

STEP 2: Synthesis of hexagonal antimonene nanosheets:

In a 3-neck flask with a septum, a condenser and a nitrogen adapter OA (0.5 mL) and ODE (4.0 mL) were added. Similar to the Sb-DDT precursor the OA and ODE mixture was degassed at 110°C for 30 minutes. Afterwards, the apparatus was flushed with argon and a bubbler was put on the condenser, so that pressure equalization can take place. Thereafter, the reaction mixture was heated to 300°C, whereupon Sb-DDT (1 ml) was injected. After 10 s at 300 °C the flask was cooled down rapidly using a water-ice bath. To obtain clean and well-defined hexagons, a solvent exchange was performed to remove all residues of unreacted chemicals. The sample was centrifuged (10000 rpm for 5 min) and the solvent mixture was exchanged with $CHCl_3$. This was repeated two additional times to obtain the Sb NS in $CHCl_3$.

1.2.2. Fluorination with CF_4 plasma:

New Si/SiO_2 wafers were cleaned with propan-2-ol, whereupon a Sb NS dispersion was dropcasted onto the wafers. After the solvent evaporated, the wafer was washed three times with $CHCl_3$. For a complete removal of the solvents the wafers were dried in the vacuum oven at 70 °C for 1 h. Afterwards the samples were sealed and transported to the "Fraunhofer-Institut für Integrierte Schaltungen IIS" in Erlangen, for the subsequent CF_4 plasma treatment.

2. Instrumentation

Atomic force microscopy:

A Bruker Dimension Icon microscope in scan-assist-mode was used. A Bruker ScanAsyst-Air silicon tip with a diameter of around 10 nm was used to obtain images with a resolution of 512x512 or 1024x1024 pixels. The Gwydion software was used for post-processing like flattening, image correction and extraction of the corresponding thicknesses and lengths.

Raman spectroscopy:

Raman spectroscopic characterization was carried with the help of a HoribaLabRAM Aramis confocal Raman microscope. The system is equipped with laser of the wavelength λ = 532 nm The laser spot size was ca. 1 µm (objective: Olympus LMPlanFI 100x, NA 0.80) and the grating was 1800 or 600. The system has a filter wheel that can reduce the laser energy from 100 % to 50 %, 25 %, 10%, 5%, 3.2%, 1%, 0.1% or 0.01%. For this characterization the laser energy was reduced to 1%.

Plasma generators:

For the MIP method a Tepla GIGAbatch 360 microwave oven was used with a constant CF4 gas flow of 200 Sccm at 0,8 mbar. The energy of the generator was 800 W, and the plasma treatment was performed for 30 s.

For the RIE method an Oxford PlasmaPro 100 ICP generator was used. Using a pressure of 0.05bar a CF4 gas flow of 80Sccm was applied. The energy of the generator was 800 W, and the plasma treatment was performed for 30 s. In addition, the inductively coupled plasma was set to 400 W.

X-ray Photoelectron Spectroscopy:

X-ray photoelectron spectroscopy (XPS) measurements were performed in an ultrahigh vacuum system ESCALAB210 (base pressure 1.0 × 10−10 mbar) from Thermo VG Scientific. Photoelectrons were excited by using the Al Kα line (1486.6 eV). All spectra were referred to the Fermi level.

Samples was prepared by drop casting the Sb dispersion on an unpolished gold film, whereupon the sample was cleaned by sonicating it in 2-propanol for 2 min. After drying in air the sample was transferred to an argon atmosphere. XPS of functionalized and pristine Sb were collected at room temperature, using monochromatized Al Kα radiation to collect the data. For high resolution scans a pass energy of 10 eV was used by a base pressure of ~ 10 mbar. The binding energies were calibrated to the binding energy of Au 4f7/2 at 84 eV. Pseudo-Voigt profiles were used to fit the obtained data, which is a mixture of Lorentzian and Gaussian with 40 % Gaussian contribution.

Computational details

DFT calculations were performed using SIESTA code.[3] General gradient approximation (GGA) in combination with PBE functional were used to describe the exchange correlation energy.[4] We used a double-ζ basis set for all atoms and core electrons were described using norm-conserving Troullier−Martins fully relativistic pseudopotentials. Spin-orbit coupling effects were considered as implemented in SIESTA.[5] A real-space mesh cutoff of 400 Ry and a 4x4x1 Monkhorst−Pack k-point mesh was used in all the calculations.

3. DFT calculations

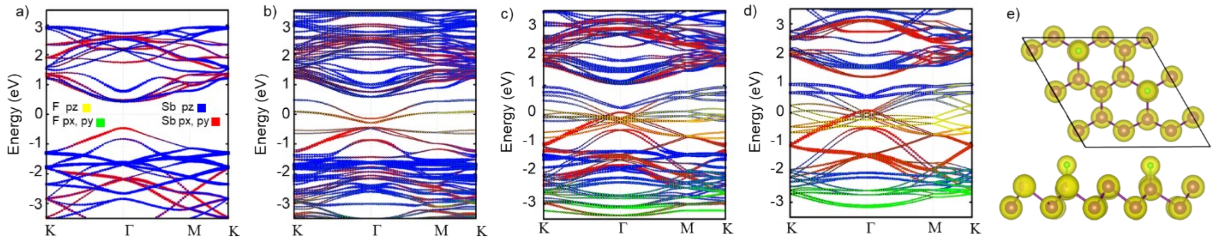

Figure S1: Band structure with atomic and orbital contributions for a) Sb_{18}, b) $Sb_{18}F_3$, c) $Sb_{18}F_6$, and d) $Sb_{18}F_9$ monolayer; e) Top and side view of $Sb_{18}F_2$ monolayer charge density (bottom). Isosurface is set to 0.08 e/Å3. Colour code: purple (Sb) and green (F).

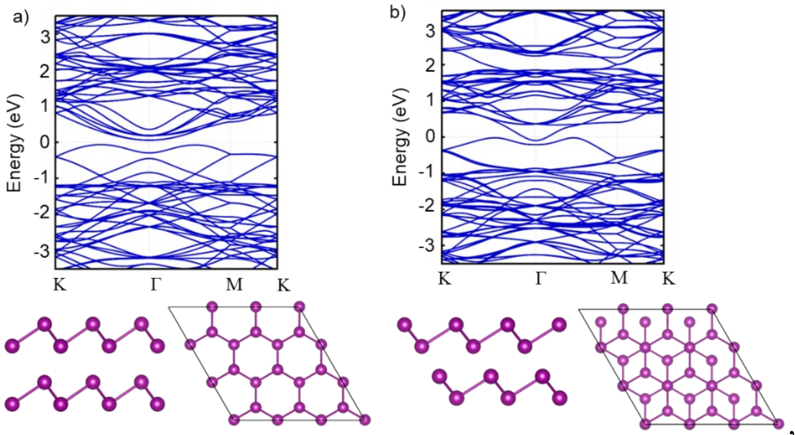

Figure S2. Electronic band structure, side and top views of β-Sb bilayer with different stacking configurations. a) AA stacking and b) AB stacking.

4. Sb hexagons characterization

A representative population of the antimonene flakes obtained in this work is shown at Figure S3. These flakes were obtained through the procedure previously developed at our group[2] and one can observe their perfect hexagonal structure. The hexagons exhibit lateral dimensions in the range of 0.6-1.4 microns and heights in the range of 7-20 nm, see Figure S3 top. A typical flake obtained with this protocol observed under the optical microscope and the atomic force microscopy (AFM) analysis is shown in Figure S3 middle. The antimonene flake exhibits a flat and relatively smooth surface. The height profile extracted from the AFM data displays a thickness of 13 nm with a size at the marked white line (see Figure S3 middle left) of 1.4 μm. Besides, the structure of the antimonene flakes was confirmed using Raman microscopy. Thus, spatial mapping of the A_{1g} band provided the Raman map shown in Figure S1 bottom left, which matches perfectly with the optical and AFM images. Finally, the mean Raman spectrum of the whole antimonene hexagon was obtained, see Figure S3 bottom right. This spectrum shows two well-defined vibrational modes at 125 and 161 cm^{-1} that correspond to the in-plane (E_g) and the out-of-plane (A_{1g}) vibrations of antimony, respectively.[6]

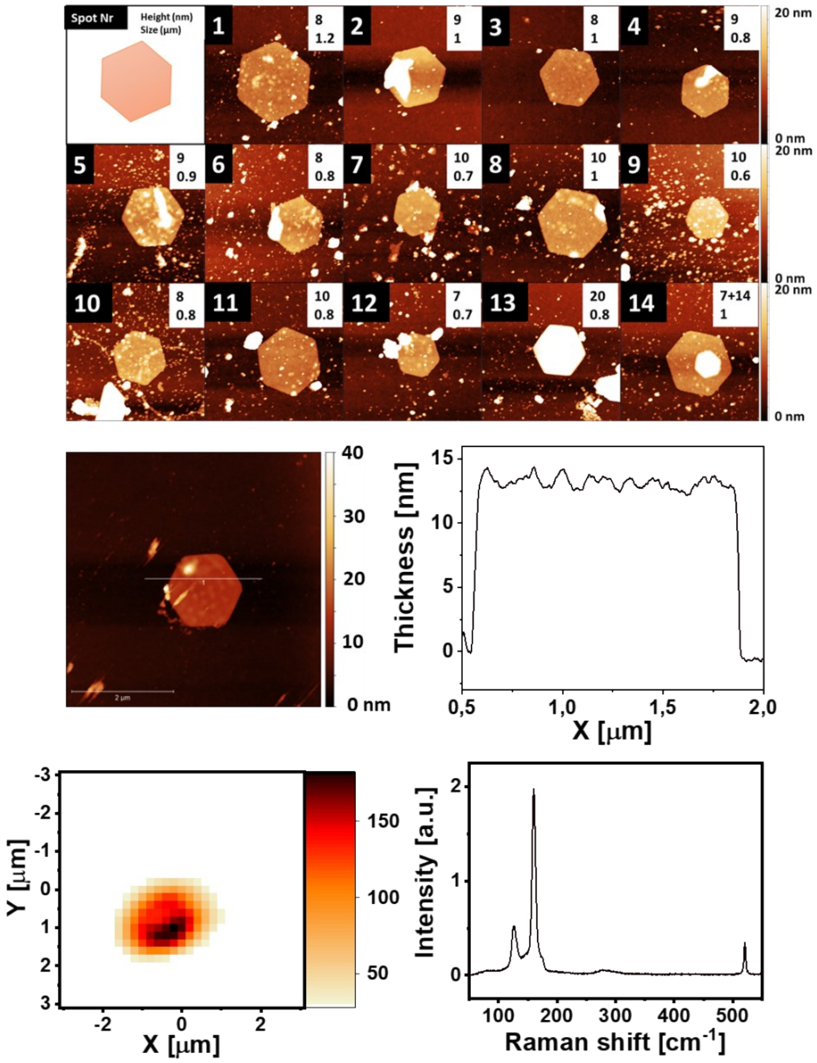

Figure S3: Optical images of a representative population of antimonene hexagonal flakes indicating their height and their lateral dimensions (top). AFM profile of a representative individual antomonene hexagonal flake with a scale bar of 2 μm (middle left) and its thickness profile (middle right). Raman mapping of A_{1g} (150 cm^{-1}) intensity (bottom left) and mean Raman spectrum of the Sb hexagon on the regions of 50–550 cm^{-1}.

5. XPS characterization

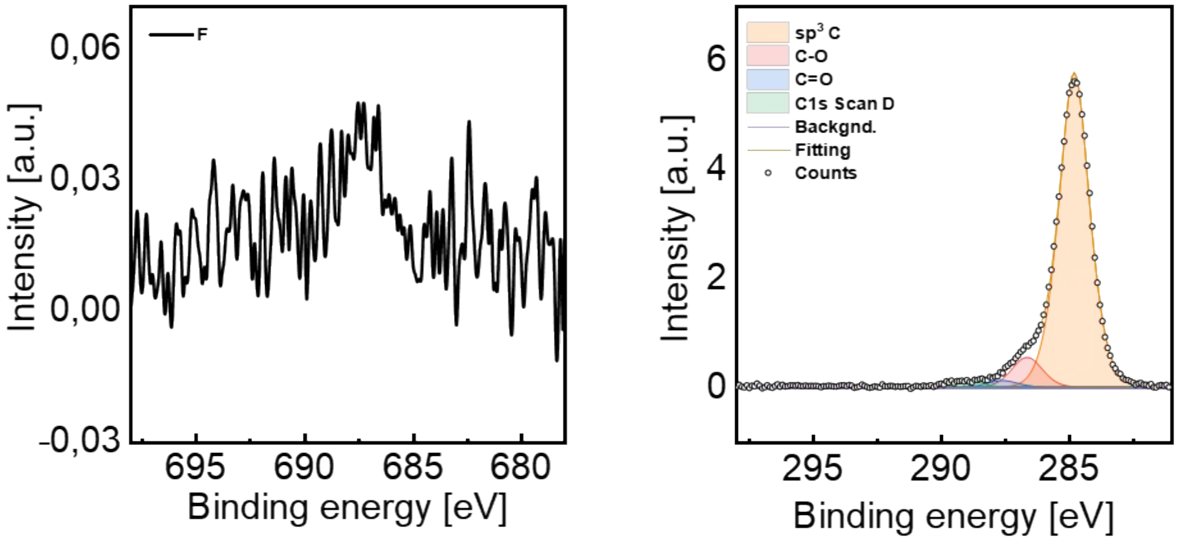

Figure S4: XPS spectra of pristine Sb hexagons at the fluorine (left column) and the carbon (right column) binding energy regions.

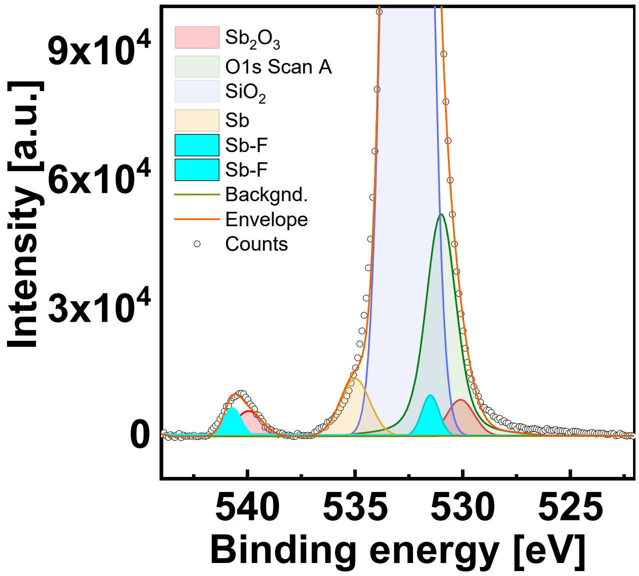

Figure S5: XPS spectra of Sb hexagons treated with RIE at the antimony and oxygen binding energy region.

Table S1: Comparison among Pristine Sb hexagons, Sb hexagons treated with MIP and Sb hexagons treated with RIE: X-ray photoelectron spectroscopy (XPS) binding energies, morphology and success of the functionalization.

		**Pristine Sb hexagons**		**Sb hexagons treated with MIP**		**Sb hexagons treated with RIE**	
**XPS**	**C**	Csp^3	284.8 eV	Csp^3	284.8 eV	Csp^3	284.8 eV
		C-O	286.6 eV	C-O	286.4 eV	C-O	286.4 eV
		C=O	287.6 eV	C=O	287.5 eV	C=O	287.7 eV
				O-C=O	289.4 eV	O-C=O	289.3 eV
				C-F	288.5 eV		
				$C\text{-}F_2$	291.0 eV	$C\text{-}F_2$	291.8 eV
				$C\text{-}F_3$	293.2 eV		
				$C\text{-}F_4$	295.1 eV		
	**F**			F-C	688.5 eV	F-C	689.1 eV
						F-Si	686.5 eV
						F-Sb	685.2 eV
	**Sb**	-		-		Sb_2O_3	530.1 eV<br>540.0 eV
						Sb-Sb-F	531.5 eV<br>540.7 eV
						Sb	535.0 eV
**MORPHOLOGY**		Lateral dimensions: 0.6-1.4 μm<br>Heights: 7-20 nm		No modification (respective to pristine)		No modification (respective to pristine)	
**FUNCTIONALIZATION SUCCESS**		**NO FUNCT.**<br>**(No Sb-F bond)**		**NO FUNCT.**<br>**(No Sb-F bond)**		**SUCCESFUL FUNCT.**<br>**(Sb-F bond formation)**	

6. References

1. Peng, L., Ye, S., Song, J. & Qu, J. Solution-Phase Synthesis of Few-Layer Hexagonal Antimonene Nanosheets via Anisotropic Growth. *Angew. Chem. Int. Ed.* **58**, 9891–9896 (2019).

2. Torres, I. *et al.* Continuous-Flow Synthesis of High-Quality Few-Layer Antimonene Hexagons. *Adv. Funct. Mater.* **31**, 2101616 (2021).

3. Soler, J. M. *et al.* The SIESTA method for *ab initio* order- *N* materials simulation. *J. Phys. Condens. Matter* **14**, 2745–2779 (2002).

4. Perdew, J. P., Burke, K. & Ernzerhof, M. Generalized Gradient Approximation Made Simple. *Phys. Rev. Lett.* **77**, 3865–3868 (1996).

5. Cuadrado, R. & Cerdá, J. I. Fully relativistic pseudopotential formalism under an atomic orbital basis: spin–orbit splittings and magnetic anisotropies. *J. Phys. Condens. Matter* **24**, 086005 (2012).

6. Wang, X., Kunc, K., Loa, I., Schwarz, U. & Syassen, K. Effect of pressure on the Raman modes of antimony. *Phys. Rev. B* **74**, 134305 (2006).
